# Mapping of prehaustorial resistance against wheat leaf rust in einkorn (*Triticum monococcum*), a progenitor of wheat

**DOI:** 10.3389/fpls.2023.1252123

**Published:** 2023-10-23

**Authors:** Mathieu Deblieck, Frank Ordon, Albrecht Serfling

**Affiliations:** Institute for Resistance Research and Stress Tolerance, Julius Kühn-Institute (JKI), Federal Research Centre for Cultivated Plants, Quedlinburg, Germany

**Keywords:** *Triticum monococcum*, leaf rust, hypersensitive response, grain yield, quantitative resistance

## Abstract

Wheat leaf rust (*Puccinia triticina*) is one of the most significant fungal diseases of wheat, causing substantial yield losses worldwide. Infestation is currently being reduced by fungicide treatments and mostly vertical resistance. However, these measures often break down when the fungal virulence pattern changes, resulting in a breakdown of vertical resistances. In contrast, the prehaustorial resistance (phr) that occurs in the einkorn–wheat leaf rust interaction is race-independent, characterized by an early defense response of plants during the prehaustorial phase of infestation. Einkorn (*Triticum monococcum*) is closely related to *Triticum urartu* as a progenitor of wheat and generally shows a high level of resistance against leaf rust of wheat. Hence, einkorn can serve as a valuable source to improve the level of resistance to the pathogen in future wheat lines. In particular, einkorn accession PI272560 is known to exhibit a hypersensitive prehaustorial effector triggered immune reaction, preventing the infection of *P. triticina*. Remarkably, this effector-triggered immune reaction turned out to be atypical as it is non-race-specific (horizontal). To genetically dissect the prehaustorial resistance (phr) in PI272560, a biparental F_2_ population of 182 plants was established after crossing PI272560 with the susceptible *T. boeoticum* accession 36554. Three genetic maps comprising 2,465 DArT-seq markers were constructed, and a major QTL was detected on chromosome 5A. To locate underlying candidate genes, marker sequences flanking the respective QTL were aligned to the *T. urartu* reference genome and transcriptome data available from the parental accessions were used. Within the QTL interval of approximately 16.13 million base pairs, the expression of genes under inoculated and non-inoculated conditions was analyzed via a massive analysis of cDNA (MACE). Remarkably, a single gene located 3.4 Mbp from the peak marker within the major QTL was upregulated (20- to 95-fold) after the inoculation in the resistant accession in comparison to the susceptible *T. boeoticum* accession. This gene belongs to a berberine bridge enzyme-like protein that is suspected to interact on the plant surface with glycoside hydrolases (GH) secreted by the fungus and to induce a hypersensitive defense reaction in the plant after fungal infections.

## Introduction

Wheat leaf rust (*Puccinia triticina*) belongs to the economically most important obligate biotrophic pathogens of wheat (Bolton et al., 2008a; Bolton et al., 2008b; Kolmer, 2013). It is the causative agent of leaf rust, the most common rust of wheat worldwide reducing number of grains and thousand-grain weight, resulting in yield losses of up to 60% (Bancal et al., 2007; Bolton et al., 2008b; Kolmer, 2013). Uredinia, the typical leaf rust fruiting bodies formed during the asexual life cycle, occur on the upper surface and bottom side of leaves on susceptible wheat cultivars with a diameter of up to 1.5 mm (Kolmer, 2005; Kolmer, 2013). These uredinia harbor dikaryotic uredospores of approximately 20 µm. When the leaf epidermis ruptures, the orange-yellow uredospores are spread by the wind to infect new host plants under favorable conditions (i.e., 10°C to 20°C and high humidity) (Bolton et al., 2008a).

At the beginning of a successful penetration process, uredospores germinate 4 to 8 hours after inoculation (hai) and form a germ tube and an appressorium over stomata cells (Bolton et al., 2008a). Within 12–24 h after the formation of the anppressorium, an infection vesicle is generated, and infection hyphae grow between parenchymal cells and form haustorial mother cells (hmc) on the cell walls of mesophyll cells (Bolton et al., 2008b). Next, 24 h after the formation of an appressoria, haustoria start to develop (Serfling et al., 2016). They penetrate the host cells and generate an extrahaustorial membrane (Bolton et al., 2008b).

Race-specific resistance is known to be effective “posthaustorial”, thus after the formation of haustorial mother cells (Niks, 1988; Niks, 1991). Hypersensitive cell death is triggered by a gene-for-gene recognition of effectors (Ji et al., 2022, Kumar et al., 2021) and is contrary to adult plant resistance (APR) already active at the seedling stage. Moreover, race-specific resistance is vulnerable to breakdown by virulent races, which occurs after a mutation of an elicitor or the interacting resistance gene in the host. More than 100 leaf rust resistance genes (*Lr-*genes) have been described; however, only a few are carried by cultivars due to linkage drag and undesired agronomic properties (Mapuranga et al., 2022). The lifespan of such vertical/qualitative resistance carried by cultivars was calculated by Mapuranga et al. (2022) at 3 to 5 years. Hence, a horizontal/race-independent resistance that has the character of nonhost resistance to leaf rust as a host-specific pathogen could be more durable. This nonhost–pathogen interaction was exemplarily described for barley—*P. triticina* (Jafary et al., 2008; Haghdoust et al., 2021) and wheat—*Blumeria hordei*, *P. hordei*, and *Magnaporthe oryzae* (Delventhal et al., 2017). Nonhost interactions result in prehaustorial resistance (phr) and have also been observed in *T. monococcum–P. triticina* interactions (Serfling et al. 2016; Anker et al. 2001; Anker and Niks, 2001). Various studies have shown a different expression of pathogenesis-related (PR-) genes, peroxidases, chitinases, peroxidases, and beta 1,3 glucanase within the first 24 hai. However, the inheritance of nonhost resistance of *T. monococcum* to *P. triticina* has not yet been genetically analyzed.

*Triticum monococcum* accession PI272560 (Serfling et al., 2016) shows complete and nonhost resistance to six investigated leaf rust (**Tables S1A, B**) races (Serfling et al., 2016) and one leaf rust race tested by Niks (1991). This resistance was previously identified as a phr in which leaf rust develops few or no haustorial mother cells after infection because their formation is prevented by an effective defense reaction of the plant (Anker et al., 2001). Serfling et al. (2016) indicate an increased level of phenolic substances, peroxidase, and chitinase activity at the site of infection and pathogenesis-related genes in the first 24 hai in comparison to the partially susceptible *T. boeoticum* accession (36554). These results demonstrate transcriptome alterations and resistance mechanisms in the background of phr in PI272560. However, the genetic background and the inheritance of this resistance were not elucidated up to now. Therefore, this study aims to identify the genomic regions and candidate genes involved in the phr of *T. monococcum* based on microscopic analysis of fungal development, the defense reaction, and visual rating in a biparental F_2_ mapping population derived from *T. monococcum* accession PI272560 and T. boeoticum accession 36554. These investigations are complemented by data from transcriptome analysis by massive analysis of cDNA ends (MACE) of the parental accessions to be mapped into QTL regions.

## Materials and methods

### Plant material

The *T. monococcum* accession PI272560 (*T*. *monococcum* var. *monococcum* variety “Ungarn white”) (Anker et al., 2001) and the partially susceptible accession 36554 (*T. boeoticum* spp. *thaoudar* var. *reuteri*, variety “Angora”) (Anker et al., 2001; Serfling et al., 2016) were obtained from the gene bank of the Leibniz Institute of Plant Genetics and Crop Plant Research (IPK, Gatersleben, Germany) and the National Plant Germplasm System (NPGS) of the United States Department of Agriculture (Aberdeen, ID, USA). Einkorn accession 36554 has previously been identified as one of the most susceptible to wheat leaf rust by Lind (2005). After crossing, the resulting F_2_ seeds were germinated on moist filter paper in petri dishes and transferred to pots with a size of 11 cm × 11 cm (height and width), filled with soil (Archut-Fruhstorfer Erde, HAWITA, Oldenburg Germany). Cultivation was conducted at 80% ± 10% humidity, at 20°C ± 2°C, and at a light intensity higher than 300 ± 15 μmol under daylight conditions (16 h). The resistant accession PI272560 was used as a pollinator. Successful crossing was confirmed in F_2_ generation by phenotyping and genotyping of seedlings.

### Leaf rust isolates, inoculation, microscopy, and phenotyping by visual rating

Ten-day-old 182 F_2_ plants were inoculated in a settling tower according to Hoogkamp et al. (1998). For that purpose, 3 mg of uredospores from single-spore isolate wxr77 was applied together with 2 mg of dry powdered clay to the parental accessions. This isolate originated from a collection of Nover and Lehmann (1967) (Serfling et al., 2016). Isolate wxr77 was also used for the inoculation of F_2_ progenies. Uredospores were multiplicated on leaves of the wheat variety Borenos. Then, 72 hai, fungal structures were stained with Calcofluor White M2R (Sigma Aldrich Chemie GmbH. Taufkirchen, Germany) as described by Rohringer (1977). Pictures were taken using an Axioskop 50 microscope and an Axiocam MRc camera connected to the software package Axiovision 4 (Carl Zeiss AG, Jena, Germany), using the filter set 02 (excitation filter G 365, beam splitter FT 395, and barrier filter LP 420). Autofluorescence of plant tissue was recorded using the filter set 05 (excitation filter BP 400-440, beam splitter FT 460, barrier filter LP 470) according to Serfling et al. (2016).

Three leaf segments from the middle of the third youngest leaf were taken for microscopic analysis. Ten infection sites were examined microscopically on three leaf segments per genotype so that haustorial mother cells from a total of 30 infection sites were analyzed at 48 and 72 hai. To assess the generation of uredospore pustules in relation to the investigated leaf area, pictures were taken using a stereo microscope (Stemi, 2000; Carl Zeiss, Jena, Germany) in combination with the digital camera Axiocam MRc and its software package Axiovision 4 (Carl Zeiss AG, Jena). Ten days after inoculation (dai) when the generation of uredospore pustules on the leaves was completed, macroscopic infection resistance was estimated according to McIntosh et al. (1995). This rating system allows the classification as “immune” (rated as “0”), “very resistant” (rated as “;”), “resistant” (rated as “1”), “moderately resistant” (rated as “2”), “moderately resistant to moderately susceptible” (rated as “3”), and “susceptible” (rated as “4”) in resistance testing of wheat to leaf rust. The letter “N” has been used to indicate a high degree of necrosis on leaves. However, in order to be able to calculate rating data for QTL analyses, the ratings were changed as follows: 0 (0),;, 1 (1, N), 2 (2), 3 (3), 4 (4).

### DNA extraction, genotyping, and genetic map construction

About 1 µg of purified DNA from leaf samples of each F_2_ plant was extracted according to Stein et al. (2001) and sent to the Diversity Arrays Technology (DArT) Lab (Bruce, Australia, https://www.diversityarrays.com/) for DArT-seq analysis (https://www.diversityarrays.com/services/dartseq/). DArT-seq is an efficient genotyping-by-sequencing platform, based on restriction enzyme-mediated genome complexity reduction and sequencing of the restriction fragments (Edet et al., 2018). Codominant DArT-seq SNP markers were scored with a “0” (reference allele homozygote), “1” (SNP allele homozygote), and “2” (heterozygote: presence of both reference and SNP alleles), while dominant DArT-seq markers were scored in a binary fashion, with “1” and “0” representing presence or absence variation (PAV) of the restriction fragment with the marker sequence (Kilian et al., 2012). For the selection of markers, grouping, and construction of the genetic map, JoinMap (Van Ooijen, 2006) was applied. Monomorphic markers were removed. Subsequently, data files were converted into an “abh” matrix (codominant DArT markers), “db” matrix (SNP alleles from maternal parent 36554) and an “ac” matrix (SNP alleles from paternal parent PI272560).

All markers were analyzed for their goodness of fit to the appropriate expected segregation ratios (1:2:1, 1:3, or 3:1) using the chi-square (*χ* ^2^) test (Olivera et al., 2013). All segregations showing a significant *χ* ^2^ test at a level of 0.05, where the threshold for one degree of freedom (df) was 2.7 (ac; bd matrix) and that for 2 df was 4.59 (abh matrix), were excluded. Markers with >10% missing information and a significant segregation distortion (alpha 0.05) were removed. To avoid repulsion effects of dominant and codominant markers, different strategies were developed to cope with this issue (Knapp et al., 1995; Peng et al., 2000; Mester et al., 2003). Therefore, three different genetic maps were constructed according to Edet et al. (2018), that is, for codominant and dominant DArT-seq markers, respectively. Linkage groups were generated based on the population node at a stringency of the threshold value that enabled the formation of seven groups according to the number of chromosomes. Genetic distances were calculated according to Kosambi (1943). By applying a standard BLASTN search against the *T. urartu* genome according to Ling et al. (2018), unique positions of the DArT-Seq markers on the corresponding chromosomes were identified. Markers that could not be grouped into chromosomes by the Joinmap function “Group” were excluded from further analysis. In case that the orientation in maps was not the same after comparison of physical and genetic positions, the orientation of the corresponding linkage group was swapped.

### Phenotypic data, statistical analysis, and QTL detection

Before QTL detection, phenotypic data were prepared as follows: Outliers were filtered out, if they were higher or lower than plus or minus three times the standard deviation of the mean. Then, quantile–quantile (QQ) plots were created to remove non-normal distributed data at the QQ plot residuals manually. Shapiro–Wilk tests (SW-tests, Shapiro and Wilk, 1965) were applied to confirm normal distribution. Abnormally distributed data were (log-) transformed, if possible. In that, the transformed visual rating scale nomenclature (McIntosh et al., 1995) was used for QTL analysis. Finally, a single-trait QTL simple interval mapping (SIM) analysis was conducted with MapQTL 5.0 by interval mapping (Van Ooijen, 2006; Kyazma, Wageningen, Netherlands).

To detect the respective thresholds of statistically significant LOD scores, permutation tests (1,000 repeats) were applied as previously described by Van Ooijen, 2006. Level of significance is needed to prove a QTL, and a relative cumulative count of 1 − 0.05 = 0.95 according to a *p*-value of 0.05 was used. Results from a MACE data of accessions PI272560 and 36554 8 hai, 16 hai, and 24 hai and a control variant without any inoculation (Serfling et al., 2016) were used to improve candidate gene identification. These MACE data comprised sequence tags of PI272560 and Tb36554 samples obtained 8 hai, 16 hai, and 24 hai with leaf rust isolate wxr77. MACE data from 8 hai, 16 hai, and 24 hai and in parallel data of the non-inoculated control samples were available (Serfling et al., 2016). To detect differences of the expression between the parental accessions, the relative expression values (REVs) of each MACE tag were calculated as follows.


$$\text{REV}1\;\;\;=\;\;\;\;\;\frac{\left(\frac{\left(\;\text{Pi}272560\;\text{MACE}\;\text{tag}\;\right)}{\sum^{}\text{MACE}\;\text{of}\;\text{PI}272560\;\text{sample}}\;\right)}{\begin{array}{c} \left(\frac{\left(36554\;\text{MACE}\;\text{tag}\;\right)}{\sum^{}\text{MACE}\;\text{of}\;\text{Tb}36554\;\text{sample}}\right) \end{array}}$$

$$\;\;\;\text{REV}2\;\;=\;\;-1*(\frac{\left(\frac{\left(36554\;\text{MACE}\;\text{tag}\;\right)}{\sum^{}\text{MACE}\;\text{of}\;\text{the}\;36554\;\text{sample}}\;\right)}{\left(\frac{\left(\text{Pi}272560\;\text{MACE}\;\text{tag}\right)}{\sum^{}\text{MACE}\;\text{of}\;\text{PI}272560\;\text{sample}}\;\right)\;}$$

By dividing the number of a MACE tag within a specific sample through the sample’s total MACE number, the sampling effect was eliminated (Serfling et al., 2016). REV1 describes the relative expression of a specific MACE tag of PI272560 vs. Tb36554, while REV2—*vice versa*—describes the relative expression of a specific MACE tag of Tb36554 vs. PI272560 at the same time segment and the same variant (inoculated or not inoculated). Since REV1< 1 values equal to REV2< −1 and REV2 values > −1 equal to REV1 > 1, only REV1 > 1 and REV2< −1 were considered. To anchor the MACE tags to the *T. urartu* genome (taxid 4572), a MEGABLAST (Morgulis et al., 2008) search against all *T. urartu* genes was applied with an exception cutoff of 0.001 (E-value). Finally, based on scores for homology, the best BLAST-hit of each MACE was considered, if the percentage identity scores were above 95% and matched a gene on the *T. urartu* chromosome 5A (Ling et al., 2018).

## Results

### Genetic map construction

After crossing PI272560 and the partially susceptible 36554, 182 F_2_ plants were genotyped using the DArT-seq array based on Jing et al. (2009). Out of 2,138 dominant markers and 7,984 codominant markers, after excluding monomorphic, as well as non-grouped markers and markers showing minor allele frequency<5% or a high number of missing data, 2,465 markers were included in three genetic maps. One map contains codominant SNP markers and two maps contain dominant markers for both parental alleles. The three different genetic maps have a size of 1,341.45, 945.29, and 1,046.52 cM (**Table 1**).

**Table 1:** Distribution, positions, and number of markers of mapped DArT-seq markers within the three genetic maps (size is shown in cM) for dominant and codominant markers, respectively.

Chr.	Codominant markers			Dominant markers for the PI272560 allele			Dominant markers for the 36554 allele			Physical size
	Positions	Markers	Size	Positions	Markers	Size	Positions	Markers	Size	
1A	225	74	182.55	299	239	120.57	306	199	169.18	584,104,260
2A	223	69	185.38	233	155	117.86	215	118	143.88	753,704,009
3A	251	48	222.88	281	148	184.45	294	137	197.28	747,003,405
4A	171	36	151.44	226	120	147.42	156	61	112.21	619,557,940
5A	206	78	167.39	201	135	112.44	168	107	97.41	661,454,495
6A	184	35	166.87	294	198	153.42	192	76	134.97	575,711,938
7A	296	54	264.94	288	232	109.13	290	146	191.59	719,654,360
Sum	1,556	394	1,341.45	1,822	1,227	945.29	1,621	844	1,046.52	

As a comparison, the physical size of *T. urartu* chromosomes is shown in base pairs (bp).

Furthermore, the sequenced *T. urartu* genome (Ling et al., 2018) was used to anchor the DArT-seq markers to base pair positions of the respective physical chromosomes (pseudomolecules). In general, the arrangement of markers in the genetic and physical maps was comparable. However, in a few cases, the chronology of markers differed, e.g., at the tips of the chromosomes or inside inverted chromosome fragments. The respective genetic maps, phenotypic data, and QTL LOD values along the chromosomes are summarized in **Table S2**.

### Phenotypic data and QTL detection

**Figure 1:**
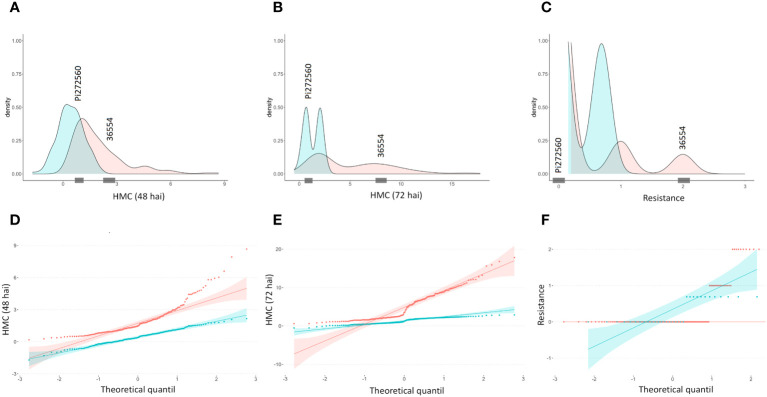
Density plots and quantile–quantile plots of microscopically counted haustorial mother cells 48 and 72 hai and macroscopic resistance data. Macroscopic resistance data were obtained 10 days after inoculation (dai) after the generation of uredospore pustules. Original and (log-)transformed data are colored in blue and red, respectively. From 182 F_2_ genotypes, the distribution of the haustorial mother cell number **(A)** 48 h, **(B)** 72 h, visual rating, and **(C)** 10 days after the inoculation is shown. Quantile-quantile plots of HMC data **(D)** 48 hai, **(E)** 72 hai and **(F)** rating data 10 dai. Observed hmc and rating values of the parental lines are represented on the x-axis by gray markings.

After carrying out the SW-test, it became apparent that neither hmc data (48 hai and 72 hai) nor visual rating data (**Table S3**) are normally distributed, but right skewed. While a (log-) transformation to normal distribution succeeded to transform the 48 hai hmc data to normal distribution, the hmc data (72 hai) and macroscopic data remained non-normally distributed (**Figure 1**, **Table 2**). According to previous studies (Serfling et al., 2016), generation of hmc 48 hai differed in the amount of counted uredospore pustules per mm² in leaf tissue between 0.35 ± 0.17 of PI272560 and 2.54 ± 0.64 (36554) and 72 hai between 0.53 ± 0.05 and 11.38 ± 1.99. Seven days after the inoculation, the parental line PI272560 did not show any colonies whereas line 36554 showed 0.39 ± 0.06 uredospore pustules per mm². Phenotypes of the F_2_ population ranged between 0.18 and 8.67 hmc (48 hai), 0.87 and 16.83 hmc (72 hai), and from complete resistant (rated as “0”) to most susceptible phenotypes rated as “2”.

**Table 2:** Distribution of the haustorial mother cell number (hmc) and the rating score within the F_2_ mapping population 48 h after inoculation (hai), 72 hai, and after 10 days.

	HMC number (48 hai)	HMC number (72 hai)	Rating score
Minimum	0.18	0.60	0.00
1st Quartile	0.93	1.90	0.00
Median	1.47	3.50	0.00
Mean	1.97	5.05	0.24
3rd Quartile	2.52	7.80	0.00
Maximum	8.67	17.80	2.00

**Figure 2:**
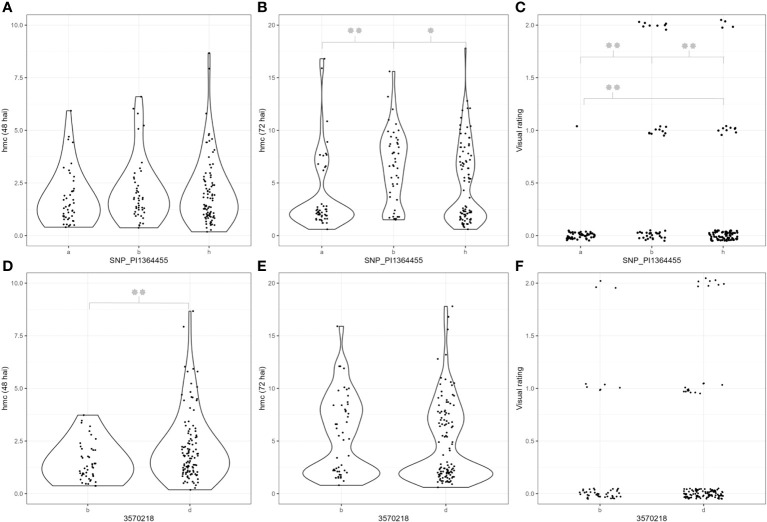
Phenotypic data of genotypes carrying different alleles of the SNP_PI1364455 and 3570218 markers. Hmc = Haustorial mother cells. The “a” and “b” alleles represent genotypes that are homozygous for the PI272560 or Tb36554 allele. “h” represents heterozygous genotypes. **(A, D)** Haustorial mother cell data 48 hai, **(B, E)** haustorial mother cell data 72 hai, and **(C, F)** resistance data. Significant differences between the respective subgroups were calculated with a two-tailed independent *t*-test. *p*-values ≤ 0.05 or 0.01 were marked with one or two stars, respectively.

None of the three different phenotypic datasets correlate significantly (α = 0.01, 0.05, Spearman test) (**Table 3**). However, analysis of visual rating and hmc data at 72 hai led to the identification of a single QTL on chromosome 5A in two of the three genetic maps with a LOD value of 12.6 (hmc 72 hai) and 4.6 after processing visual rating (**Table 4**). Aligning the flanking and peak markers of these QTLs (**Table 4**) to the *T. urartu* reference genome revealed that both the hmc (72 hai) and rating-based QTL are located within the same physical interval in the *T. urartu* reference genome (**Table 4**). Both QTLs show the same peak with the SNP marker SNP_1364455 at the tip of the QTL (**Table 4** and **Table S2**). Almost all (49 of 50) F_2_ genotypes showing the PI272560 allele were rated as completely resistant, whereas plants being heterozygous or homozygous for the 36554 allele harbor a considerably higher fraction of susceptible (rating 2 or 3) F_2_ plants (**Figure 2**).

**Table 3:** Pearson correlation between rating data, haustorial mother cell (hmc) generation at 48 hai and at 72 hai.

	Visual rating	Hmc at 48 hai	Hmc at 72 hai
Visual rating	1.00	0.03	0.07
Hmc at 48 hai	−0.14	1.00	−0.14
HMC at 72 hai	−0.03	0.07	1.00-

Significant correlations are marked by *(α = 0.1), **(α = 0.05), and ***(α = 0.01).

**Table 4:** QTL regions on chromosomes (chr.) 5A and 7A based on genetic maps calculated with dominant markers (dm) and codominant markers (cdm).

Chr.	Maps	Traits	Interval (cM)	Interval (Mbp).	PEV	Add.	Markername	Peak (Mbp)	LOD	LOD cutoff
5A	cdm	Hmc at 72 hai	73.94–103.37	418.56–481.75	10.9	−1.62	SNP_1364455	453.78	4.56	2.80
5A	cdm	Rating	61.64–120.36	386.36–501.64	26.9	−0.29	SNP_1364455	453.78	12.39	2.80
5A	dm	Hmc at 72 hai	55.77	449.77	11.0	−1.49	2326504	449.77	4.51	4.40
7A	dm	Hmc at 48 hai	188.39–191.59	1.24–3.57	60.9	1.86	1252815	701.29	13.87	13.70

QTL intervals of genetic maps (centiMorgan, cM) were compared to the physical position available from the *T. urartu* genome (million base pairs, Mbp). Using different phenotypic data as traits, the percentage of explained variance (PEV), logarithm of odds (LOD), and additive effect (add.) were calculated. cM and Mbp position of markers that directly flank the QTLs region above the LOD-cutoff value of the respective chromosome. The LOD-cutoff values were determined after a permutation test with 1,000 repeats and indicate a significance level of p = 0.05.

Complete resistance only occurs in genotypes homozygous for the PI272560 allele (**Figure 2**). Nevertheless, a positive effect of the PI272560 allele can be detected in heterozygous genotypes as they are significantly more resistant than genotypes homozygous for the 36554 allele (*χ* ^2^ test, *p*-value = 0.003). However, notably, genotypes being homozygous for the allele of the susceptible parent 36554 were not completely susceptible. **Figures 2A–C** illustrate that the PI272560 allele of SNP_1364455 is associated with a lower amount of HMC 72 hai and a significantly reduced or even absent infestation 10 dpi.

Furthermore, a possible minor QTL just above the significance threshold could be detected on chromosome 7A at 48 hai (**Table 4**). According to a *χ* ^2^ test, F_2_ plants carrying the 36554 allele of the respective peak marker 3570218 show a minor reduction of hmc compared to genotypes carrying the 36554 allele. However, no differences could be detected at 72 hai and visual rating after 10 days (**Figures 2D–F**).

### Identification of candidate genes on chromosome 5A

**Figure 3:**
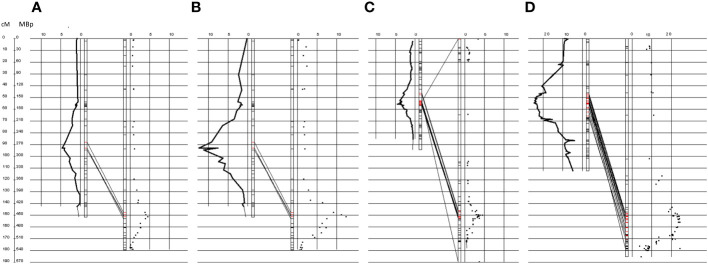
QTL for hmc (72 hai) data and resistance rated in the codominant SNPs and dominant genetic map on genetic and physical scale on chromosome 5A. Million base pair (Mbp) positions according to the sequenced *T. urartu* genome (Ling et al., 2018) are illustrated to the right side. Genetic positions of the codominant and dominant maps are illustrated to the left. Logarithm of odds (LOD) values were plotted along the chromosomes on both sides. **(A, B)** illustrate the QTLs mapped along the genetic map that was constructed with codominant markers. **(C, D)** illustrate maps that were constructed with markers that were dominant for the PI272560 or Tb36554 allele. **(A, C)** show QTLs that were obtained with macroscopic resistance data after the generation of uredospore pustules (for more details, see text). **(B, D)** illustrate QTLs that were obtained with haustorial mother cell data 72 hai (for more details, see text). Red markers indicate the presence of significant QTL LOD values, according to a permutation test (alpha = 0.05). All QTLs colocalize within the same physical region. Illustrations were created with the software GenoTypeMapper (Deblieck et al., 2020).

Independent from the different genetic maps, DArT-seq markers in general show the same order on chromosome 5A compared to the *T. urartu* genome. Furthermore, both QTL for rating data and the number of hmc at 72 hai between the flanking markers colocalize within the same physical region (**Figure 3**; **Table 4**).

After a permutation test at a level of α = 0.05, 729 genes (Ling et al., 2018) could be located based on their physical position within the QTL (trait hmc at 72 hai) on Chromosome 5A, ranging from 418.56 to 481.75 Mbp (**Table 4**, **Table S4**). Increasing the significance level from 0.950 to 0.999, the QTL region could be narrowed down to 10.6 Mbp., ranging from 443.16 to 453.78 Mbp. Within this region, the order of markers did not perfectly match the order of the physical *T. urartu* genome (**Figure 3**; **Table S5**). Therefore, based on the marker order in the genetic map, the size of the interval was increased to a region between 443.16 and 459.30 Mbp, comprising 217 genes (Ling et al., 2018; **Table S4**). MACE tags could be anchored to 117 of them.

Out of these 117 genes, 11 genes are exclusively expressed in PI272560 and 12 genes are expressed only in accession 36554 (**Table 5**). Overall, five of these genes are known to be involved in resistance reactions to fungal pathogens (**Table 5**).

**Table 5:** Exclusively expressed tags in parents PI272560 and 36554 that could be anchored within the narrowed down QTL region on chromosome 5A.

Parents	Physical position (start of sequence)	*T. urartu* gene	Hit to *T. urartu* gene (BLAST-subject sequence)	MACE tag (BLAST-query sequence)	Biotic (*) Abiotic (**)	Pident	E-value	8 h contr.	8 h inoc.	16 h contr.	16 h inoc.	24 h contr.	24 h inoc.
PI272560	443190558	2813.01	Transcription factor bHLH49	TC403977	**	97.3	0	1.63	–	–	–	–	–
	445603573	2826.01	Dof zinc finger protein MNB1A	TC420827	*/**	88.5	2E-161	–	–	0.68	–	–	–
	446396174	2830.01	Microtubule binding protein 2C	TC454483	*	97.0	0	–	–	–	0.67	–	–
	446399596	2831.01	Translation initiation factor IF-2	CA727554		97.2	1E-114	–	–	–	–	0.52	–
	449461624	2872.01	Tonoplast dicarboxylate transporter	TC417826	**	84.3	8E-71	0.82	–	–	–	–	–
	450096292	2891.01	Fasciclin arabinogalactan protein 7	TC393310	**	93.2	0	–	0.70	–	–	–	–
	452761127	2931.01	Protein LTV1 homolog	TC390467		98.9	2E-126	–	–	–	0.67	–	–
	453045990	2937.01	Cationic amino acid transporter 5	CN009021	**	93.9	0	0.82	–	–	–	–	–
	455349149	2977.01	Beta-glucosidase 30	TC433011	*/**	96.0	0	–	–	0.68	–	–	–
	457006664	2992.01	Splicing factor U2af small subunit A	CV769277	*	86.1	2E-107	–	–	–	0.67	–	–
	457034622	2998.01	Uncharacterized	TC408191		99.6	0	–	–	0.68	0.67	–	–
36554	443450603	2816.01	Queuine tRNA-ribosyltransferase catalytic subunit 1	comp41569_c0_seq1		96.7	4E-47	0.79	0.61	–	–	–	–
	448814185	2860.01	Wall-associated receptor kinase 3	TC248033	*	95.3	3E-129	0.79	–	–	–	–	–
	448825517	2861.01	Putative BPI/LBP family protein	TC262663	(*)	95.8	0	–	–	0.57	–	–	–
	450218030	2897.01	E3 ubiquitin-protein ligase UPL5	TC248540	*	92.6	2E-176	–	–	–	0.55	–	–
	450415454	2901.01	Uncharacterized	TC392852		97.8	0	0.79	–	–	0.55	–	–
	452811833	2933.01	Uncharacterized	TC402189		85.7	8E-81	–	–	–	–	1.89	–
	453106195	2941.01	Uncharacterized	TC445345		82.3	9E-93	8.67	0.61	5.71	6.59	5.67	1.12
	453280061	2945.01	Small heat shock protein, chloroplastic	BE604120	**	92.0	0	–	–	1.14	–	–	–
	453763106	2954.01	Plant UBX domain-containing protein 10	TC240157		92.9	3E-165	–	–	–	0.55	–	0.56
	455944296	2984.01	Protein EMSY 3	TC248969	*	94.0	1E-168	0.79	–	–	–	1.89	–
	457011732	2995.01	Uncharacterized	TC388510		95.6	1E-148	–	–	–	0.55	–	–
	457799874	3010.01	Phenylacetaldehyde reductase	comp41361_c0_seq1	(*)	100.0	1E-40	0.79	0.61	–	0.55	–	0.56

From tags showing a hit to genes within the *T.urartu genome*, the percentage identity (pident), expectation value (E-value), and the last six digits of T. urartu gene IDs (TuG1812Gxxxx.xx) are shown. Number of tags within variants and time segments are shown as tags per million (tpm). Genes related to abiotic or abiotic stress were marked with one or two asterisks.

**Figure 4:**
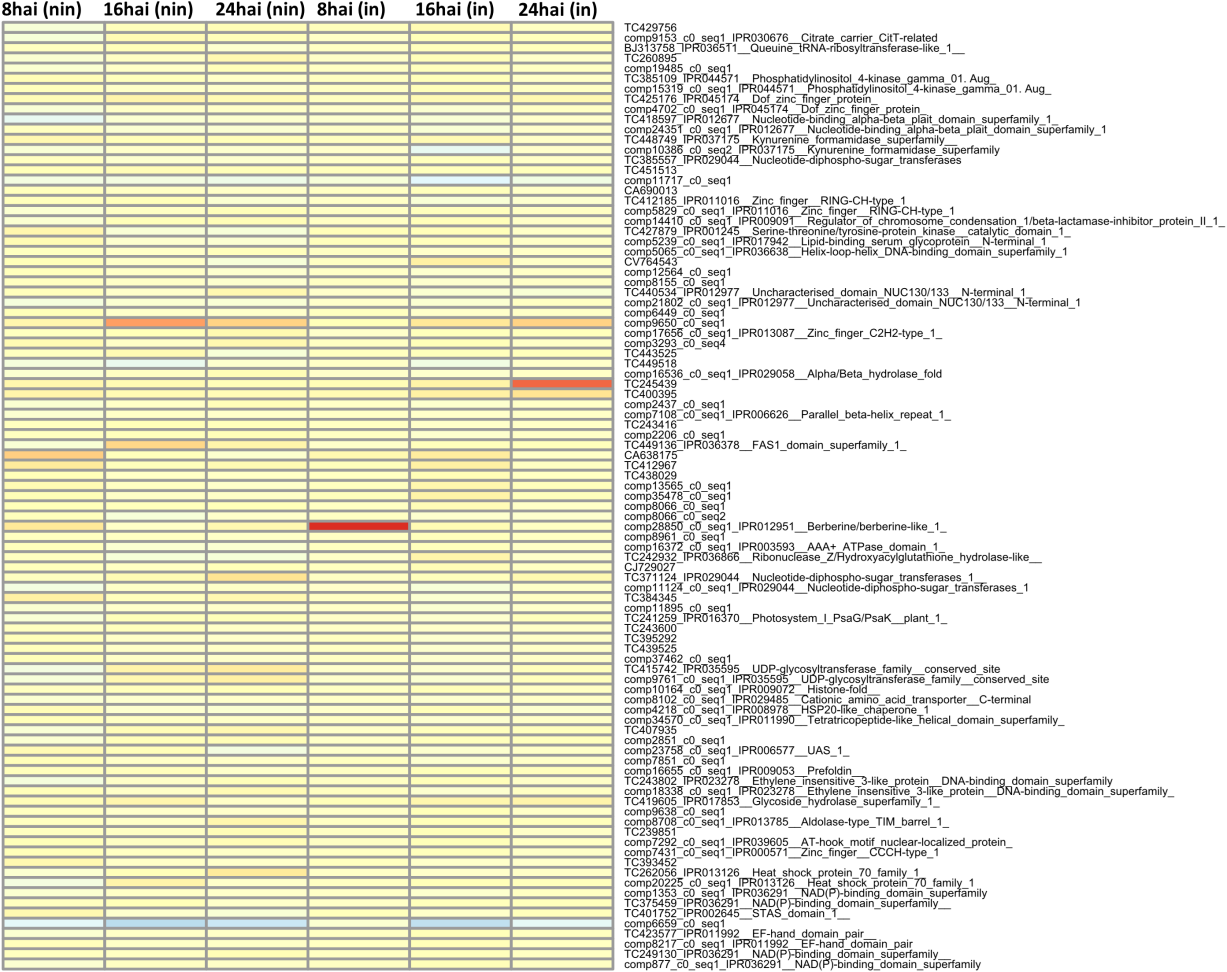
Heatmap of the most differentially expressed genes between PI272560 and 36554 in non-inoculated (columns from left to right) and inoculated variants at 8, 16, 24 hai. The number of tags per million (tpm) between PI272560 and 36554 for the same tags was compared according to the REV1 and REV2 equations. High and low expression values were colored in red and blue. For more detailed information about the REV1/REV2 values and anchoring of the MACE-tags to the genes, see the *Materials and Methods* section. Only MACE with complete expression information, i.e., under inoculated and non-inoculated conditions and at all time points, were considered.

Finally, differentially expressed MACE and their underlying genes were examined. For this purpose, the three MACE with the highest or lowest REV1 and REV2 values were identified 8–24 hai in inoculated and non-inoculated samples and summarized in **Table 6**: 8 and 14 MACE tags were quantitatively upregulated in 36554 and PI272560, respectively (**Table 6**; **Figure 4**).

**Table 6:** Differentially expressed genes 8, 16, and 24 hai within the QTL interval on chromosome 5A.

	Physical position (bp)	Gene (*T. urartu*)	InterPro	MACE tag	Pident	E-value	8 h contr.	16 h contr.	24 h contr.	8 h inoc.	16 h inoc.	24 h inoc.
Less expressed in PI272560	446399596	2831.01	RRM_dom	TC418597	98.8	0	−5.32	−4.57	−2.19	−4.62	−2.46	−2.99
	446979590	2842.01		comp11717_c0_seq1	98.3	1.94E-113	−2.79	−7.47	−5.96	−10.56	−7.57	−11.64
	449721613	2877.01		TC449518	89.9	1.25E-166	−2.42	−10.80	−2.19	−1.84	−4.04	−2.93
	449881343	2882.01	Nup186/Nup192/Nup205	comp16121_c0_seq1	93.6	5.94E-113	−4.19	3.09	1.65	−19.92	−2.83	0.00
	451320962	2914.01		TC412143	100.0	0	−4.06	−25.24	0.00	−4.98	−13.96	−9.78
	453763106	2954.01	UBX_dom	comp23758_c0_seq1	98.9	2.19E-90	3.10	1.68	−4.86	−2.81	−1.33	−1.40
	456345861	2988.01	PPC_dom	TC406617	99.8	0	−16.44	−25.24	0.00	−2.16	0.00	−8.85
	458108792	3015.01	HARBI1-like	comp6659_c0_seq1	88.6	4.16E-76	−6.01	−20.57	−12.23	−6.33	−10.67	−18.08
Higher expressed in PI272560	446784968	2834.01	UDP_glucos_trans	TC395757	95.5	9.91E-148	−2.18	11.88	0.00	−4.11	−1.23	0.00
	449473077	2873.01		comp9650_c0_seq1	95.7	1.44E-90	4.11	24.24	11.06	5.79	6.77	23.30
	449922178	2884.01		TC245439	94.0	0	3.93	3.57	1.10	8.08	4.18	47.25
	449922178			TC400395	99.0	0	4.37	3.12	1.92	10.91	4.73	18.08
	450096292	2891.01	FAS1_domain	TC449136	98.3	1.32E-83	−1.66	14.86	3.98	−2.91	1.02	2.15
	450211074	2896.01		CA638175	82.8	1.13E-57	10.34	1.02	−2.13	12.70	5.68	−1.71
	450211074			TC412967	91.7	2.56E-19	6.70	1.05	2.27	4.97	4.89	−1.23
	450211074			TC376942	98.6	7.77E-139	0.00	−1.56	−1.82	5.20	10.35	−1.21
	450397572	2899.01	Oxid_FAD_bind_/BBE	TC382880	99.0	0	7.39	−1.94	4.12	0.00	1.95	−1.70
	450397572			TC383288	95.1	0	0.00	−1.68	0.00	17.32	2.03	−1.12
	450397572			comp28850_c0_seq1	98.4	3.88E-88	7.16	−1.81	3.20	95.85	2.18	−1.15
	450415454	2901.01		TC241640	90.4	0	0.00	0.00	0.00	0.00	6.09	0.00
	452635874	2930.01	Glyco_trans_8	TC371124	97.6	0	1.18	2.97	8.51	1.03	1.02	6.44
	457231755	3004.01	Chaperone_DnaK	TC262056	94.8	0	−1.31	1.49	7.14	−1.30	−1.03	−1.49

Position of less expressed genes in PI272560 and higher expressed genes in comparison to accession 36554 are shown. From tags showing a hit to genes within the *T. urartu* genome, the percentage identity (pident), expectation value (E-value), and the last six digits of *T. urartu* gene IDs (TuG1812Gxxxx.xx) are shown. Number of tags within variants and time segments are shown as tags per million tags (tpm). Tpm detected in PI272560 and 36554 were compared to obtain the relative expression values (REV) 1 and 2 (Materials and Methods section). The position within the *T. urartu* genome is shown in base pairs (bp) for the inoculated (inoc.) and non-inoculated (contr.) variants.

**Figure 5:**
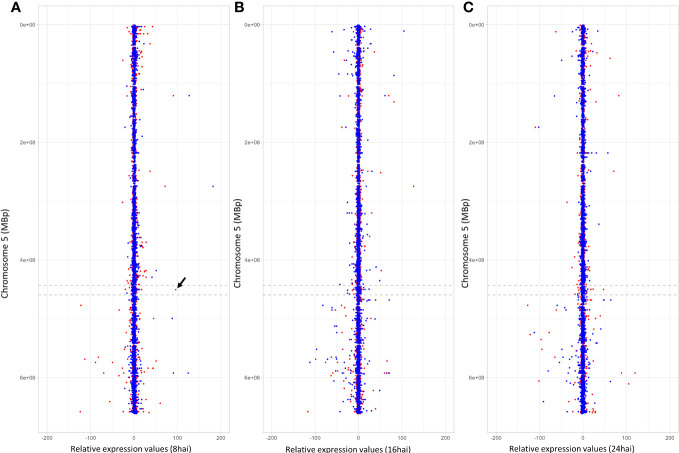
MACE expression values on chromosome 5A. Relative expression values and positions of the MACE on chromosome 5A are illustrated on the *x*- and *y*-axes. Blue and red dots represent expression values (REV), obtained under non-inoculated conditions and inoculated conditions. They describe the *x* fold expression of a MACE relative to the resistant PI272560 and were calculated according to the REV1 and REV2 equations (for more details, see text). Negative relative expression values indicate a stronger expression of the MACE tag in 36554, whereas positive values indicate a stronger expression in the resistant parent PI272560. **(A–C)** illustrate the relative expression values that were calculated for the MACE 8 hai, 16 hai and 24 hai, respectively. The arrow between the dashed lines indicates the position of the MACE with the highest differential expression in the QTL interval on chromosome 5A. This MACE tag belongs to a Berberine bridge enzyme (BBE) and is 95-fold higher expressed in the resistant accession PI272560 8 hai.

**Figure 6:**
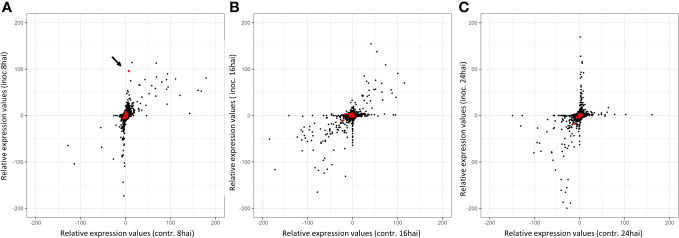
Relative expression values of MACE tags in inoculated and non-inoculated samples 8, 16, and 24 hai. Inoc = Inoculated, contr. = non-inoculated control samples. The number of tags per million (tpm) between PI272560 and 36554 for the same tags was compared (according to the REV1 and REV2 equations). Therefore, negative relative expression values indicate a stronger expression of the MACE tag in the parent 36554, whereas positive values indicate a stronger expression in the resistant parent PI272560. Red dots indicate the MACE of the genes within the QTL interval on chromosome five. Relative expression values were illustrated **(A)** 8 hai, **(B)**16 hai, and **(C)** 24 hai. The arrow highlights the position of the MACE with the highest differential expression in the QTL interval on chromosome 5A. This MACE belongs to a Berberine bridge enzyme (BBE) and is 95-fold higher expressed in the resistant accession PI272560 8 hai.

Notably, one gene (TuG1812G0500002899), encoding a berberine bridge enzyme (BBE)-like Cyn d 4, showed a 95 times higher expression at 8 hai in PI272560 than in 36554. This gene is located exactly at the peak of the QTL on chromosome 5A at 450.397 Mbp, close to the marker SNP_1364455 (**Table 6**, **Figures 3**, **5**). On a whole transcriptome level, the gene coding for a berberine bridge enzyme-like was one of the highest upregulated genes in PI272560 after inoculation in comparison to 36554 (**Figures 5**, **6**; **Table S6**). **Figures 5** and **6** illustrate the relative expression of MACE tags along chromosome 5A (Ling et al., 2018) and within the MACE data.

## Discussion and conclusion

Today, only a few known *Lr-*genes are used in wheat varieties. Most are seedling resistances, which are generally vulnerable to being broken down by races with a changed virulence pattern (Periyannan et al., 2017; Zetzsche et al., 2020). Only a few non-race-specific resistances have been described, for instance, *Lr34* and *Lr67*, which are quantitative and active at the adult plant development stages only (Hou et al., 2023). In most cases, obligate biotrophic fungal pathogens such as leaf rust are strictly host-specific. Therefore, one possibility to introduce durable leaf rust in wheat cultivars is single genes from alien species. Examples of these are the stem rust resistance genes *Sr31*, powdery mildew resistance *Pm21*, and resistance to eyespot disease resistance *Pch1* (Ellis et al., 2014; Wulff and Moscou, 2014). Alien introgressions from rye (*Secale cereale*) contribute several resistance genes for powdery mildew, leaf, stripe, and stem rust, for example, *Yr9*, *Lr25*, and *Lr26* (Johannson et al., 2020).

Hybridization between wheat and its wild relatives *Aegilops* sp., *Triticum timopheevii*, and *Thinopyrum ponticum* (Keilwagen et al., 2019) occurs naturally and is conducted during the breeding process. However, only a few rust resistances have been introgressed from diploid *T. monococcum* (*Yr34*, Chen et al., 2021; *Sr22*, Kerber and Dyck, 1973). A transfer of leaf rust resistance to wheat was reported by Hussien et al. (1997), whereas Noweiska et al. (2022) and Feuillet et al. (2003) showed that *Lr10* is conserved in grass species with similarities to *RPM1* in *A. thaliana.* Most *T. monococcum* accessions (84%) show a high level of resistance in contrast to most *T. boeoticum* accessions, including the partially susceptible *T. boeoticum* accession 36554 (Anker et al., 2001).

Hussien et al. (1997) analyzed three leaf rust resistances derived from *T. monococcum*, but did not identify their respective genetic positions. Two of them, *Lr63* and *LrTM16*, were mapped on chromosome 3A and 2A in *T. monococcum* so far (Sodkiewicz et al., 2008; Noweiska et al., 2022). Loci associated to leaf rust resistances on chromosome 5A were identified in wheat based on the Bavarian MAGIC wheat (BMW) population (QLr.jki-5A.1; QLr.cim-5AC, Rollar et al., 2021), in a DH population derived from the Canadian wheat cultivar Carberry (Bokore et al., 2021) and the cultivar Lillian (Bokore et al., 2023). Remarkably, these QTLs explain only a low level of phenotypic variance with regard to the resistance level. Furthermore, they are either effective at the seedling or adult plant stage. In contrast to that, the resistance in Pi272560 is effective at all developmental stages (**Figure 2C**). To our best knowledge, no resistance with such a high efficiency was described on chromosome 5A so far. To understand the background of the prehaustorial leaf rust resistance of *T. monococcum* accessions, the development of fungal structures was analyzed (Jacobs et al., 1996). Expression studies and microscopical analyses have been performed in different studies (Anker et al., 2001; Sánchez-Martín et al., 2012; Serfling et al., 2016). Recognition of the pathogen by resistant accessions and first defense reactions based on hydrogen peroxide accumulation and antifungal compounds could already be observed 6 hai, so that the generation of hmc, haustoria, hyphae, and pustules was inhibited (Anker et al., 2001; Serfling et al., 2016). The number of hmc at 72 hai and rating data from the F_2_ genotypes showed right-skewed or binominal distribution but no segregation known for a single gene in the background. Furthermore, due to a possible nonhost resistance of *T. monococcum* to wheat leaf rust, an identification of different loci from both parents was expected since 36554 turned out to be partially resistant against *P. triticina* as well (Serfling et al., 2016).

A QTL analysis was performed to identify the effects of both genotypes on the segregating F_2_ population. The comparison of parental lines during the first 24 hai revealed a complex defense reaction comprising various mechanisms leading to an inhibition of the infection process. In accordance with previous studies of the host–pathogen interaction, a higher expression of genes known to be involved in the reaction to leaf rust could be observed. The comparison of the PI272560 and 36554 transcriptome showed clearly that pathogenesis-related genes such as Pr1, β-1,3-glucanases (Pr2), chitinases (Pr3), peroxidases (Pr9), and other Pr-genes were significantly more expressed in PI272560 after inoculation with wheat leaf rust (**Table S7**, Serfling et al., 2016). Remarkably, however, these genes are not located within the corresponding genomic interval (Ling et al., 2018) of the QTLs detected in the course of our study (**Table 4**). Hence, transcriptome analysis provided information regarding the different expressions of genes but not necessarily about the actual resistance gene in the background of an effective (prehaustorial) resistance.

Such a resistance includes an early onset of hypersensitive response (HR), which could be triggered by genes involved in upstream metabolic processes. This study aimed to combine a MACE approach with the construction of genetic mapping and QTL detection to identify the actual candidate genes for the observed resistance against leaf rust. As expected from the partial resistance of accession 36554, a minor QTL of accession 36554 was identified on chromosome 7A (**Table 4**), resulting in a reduced number of hmc 48 hai (**Figure 2D**). However, this QTL is of limited importance as it has no impact on pustule development (**Figure 2F**). One reason is most likely the host specificity of leaf rust to wheat, while *T. monococcum* is almost a nonhost for *P. triticina* (Dracatos et al., 2018).

Complex defense reactions could be observed shortly after the inoculation with leaf rust together with a high number of differentially expressed genes. According to our results, in a nonhost reaction of wheat to barley leaf rust, Delventhal et al. (2017) could detect 2,498 differentially expressed genes, while Serfling et al. (2016) could identify 311 different defense-related genes. However, these findings offer an overview of the entire transcriptome after infection and do not narrow down regions of the genome where genes are linked to the resistance response. Consequently, an F_2_ population of 182 F_2_ plants segregating for the hmc generation 48 and 72 hai and a visual rating are suitable to detect QTL resistance.

Remarkably, one QTL that could be detected on chromosome 5A from PI272560 (**Table 4**) appears to have a major effect on the resistance level (**Figure 2**). This effect was confirmed within the three different maps and phenotypic data, the hmc (72 hai), and rating data (**Table 4**, **Figure 3**). It is conceivable that owing to the recessive nature of the resistance (**Figure 2C**), the QTL was detected more clearly in the map showing dominant 36554 markers (**Table 4**) and not in the PI272560 map since repulsion effects of the recessive alleles, within the QTL and the dominant PI272560 markers, might have hampered the QTL identification. Interestingly, the QTL explains the reduced hmc generation of approximately 26% of the phenotypic variance (**Table 4**). However, almost all—49 of 50 plants—were homozygous for the PI272560 allele of the marker SNP_1364455 and showed phr (**Figure 2C**). The QTLs’ percentage of explained variance (P.E.V.) was likely underestimated because genotypes, being homozygous for the 36554 allele of marker SNP_1364455, were not clearly susceptible (**Figure 2C**). We know that 36554 is not completely susceptible to *P. triticina* (Serfling et al., 2016); thus, this observation is unsurprising. However, the underestimation of the P.E.V. value in this study is a useful example of how the actual effect of a QTL is determined depending on the parent’s resistance properties.

The identification of the QTL region on chromosome 5A reduced the number of possible candidate genes to 217. After the selection of exclusively expressed tags (**Table 5**) only six genes are related to defense responses to biotic stress. From these genes, one, coding for a microtubule binding protein (exclusively expressed in PI272560), is known to be involved in hypersensitive response including an accumulation of hydrogen peroxide in an incompatible interaction between wheat and wheat stripe rust (Wang et al., 2016).

The highest tpm could be detected constitutively in 36554 expressed uncharacterized protein with unknown function. Other genes, for instance, coding for a Wall-associated receptor kinase 3 are described as activators of signal cascades and have been identified as involved in leaf rust resistance comparable to APR. Hence, a significant impact on resistance at the seedling stage could not be expected in our investigation. Finally, the greatest differences in expression could be observed between the parental genotypes for an AT-hook motif nuclear-localized protein 10, which was 25.2 times higher expressed in 36554 and a BBE, which was 95.9 times higher expressed in PI272560 at 8 hai than in 36554 (**Figures 4**–**6**).

BBEs belong to the flavin-dependent oxidoreductases and are described as enzymes that can interact with damage-associated molecular pattern (DAMP) and initiate hypersensitive reactions (Locci et al., 2019). Daniel et al. (2017) termed BBEs in *A. thaliana* a “treasure trove of oxidative reactions”. A higher expression of BBE was also observed in a nonhost response of barley to wheat powdery mildew (Andrzejczak et al., 2020). In the case of the coffee *Hemileia vastatrix* (coffee rust) interaction, this enzyme could be identified as a biomarker for the initial phr against the fungus (Guerra-Guimarães et al., 2015; Silva et al., 2022). According to the results of Serfling et al. (2016), during the *T. monococcum*–wheat leaf rust interaction, as a defense reaction within the first 24 hai in coffee, increased peroxidase activity and PR-like proteins, for instance, chitinases, were detected. The BBE might initiate and trigger hypersensitive cell death, as observed by Serfling et al. (2016). The early time of higher expression could also explain the phr with a strongly reduced number of haustorial mother cells in resistant genotypes of the F_2_ population (**Figures 2A–C**).

Hence, the BBE could be one key enzyme for the basal defense response (Guerra-Guimarães et al., 2015), but is also an important enzyme in (nearly) nonhost resistance (Andrzejczak et al., 2020; Wan et al., 2021). As already mentioned by Anker et al. (2001), *T. monococcum* phenotypically shows almost nonhost resistance to wheat leaf rust. A typical sign of nonhost resistance to leaf rust is an early start of effective resistance reactions before the formation of the haustorium (Anker et al., 2001). In accession PI272560, such reactions and no haustoria could be observed (Serfling et al., 2016), but a BBE could not be detected in the study due to the restriction to specific gene ontology terms. To find suitable markers and detect genes in the background of the phr, a segregating population is a prerequisite. If molecular-assisted selection (MAS), for example, the SNP marker SNP_1364455, could be applied to transmit the resistance of PI272560 in current wheat elite varieties, this would be a possibility to establish a potential nonhost resistance with very long persistence and race independence. Examples of successful alien gene transmission of resistance genes into wheat are, for instance, the stem rust resistance genes *Sr21, Sr22* (The, 1973), *Sr35* (McIntosh et al., 1995), a leaf rust resistance (Hussien et al., 1997; Anker et al., 2001), and the powdery mildew resistance gene *PmTmb* (Shi et al., 1998).

## Data availability statement

The original contributions presented in the study are included in the article/**Supplementary Material**. Further inquiries can be directed to the corresponding author.

## Author contributions

MD and AS established the plant material, performed the experiments, and wrote the manuscript. AS and FO conceived the basic idea of this research project and helped to improve the manuscript. All authors contributed to the article and approved the submitted version.
