# Supplementary material for: Mapping of prehaustorial resistance against wheat leaf rust in einkorn (Triticum monococcum), a progenitor of wheat
Source: Front Plant Sci. 2023 Oct 23;14:1252123. doi: 10.3389/fpls.2023.1252123 (PMC10626456; doi:10.3389/fpls.2023.1252123)
Supplement: Supplementary file 1 [file Table_1.docx]

**Table S1A: Macroscopic reaction and microscopically visible generation of uredospore pustules of genotypes carrying leaf rust resistance genes (*Lr-*genes) inoculated with different single spore isolates 168 hai (Serfling et al., 2016).**

|  | **Single spore isolates** | | | | | | | | | | | |  |
| --- | --- | --- | --- | --- | --- | --- | --- | --- | --- | --- | --- | --- | --- |
|  |  |  |  |  |  |  |  |  |  |  |  |  |  |
|  |  |  |  |  |  |  |  |  |  |  |  |  |  |
| **Genotype/Thatcher NIL** | **77wxR** | | **Hk1/3-04** | | **Hk12/3-01** | | **167/176WxR** | | **58WxR** | | **13/20WxR** | |  |
|  |  |  |  |  |  |  |  |  |  |  |  |  |  |
|  |  |  |  |  |  |  |  |  |  |  |  |  |  |
| **Accession/cultivar/NIL** | **Rating** | **Pustules mm^-2^** | **Rating** | **Pustules mm^-2^** | **Rating** | **Pustules mm^-2^** | **Rating** | **Pustules mm^-2^** | **Rating** | **Pustules mm^-2^** | **Rating** | **Pustules mm^-2^** |  |
| PI272560 | 0() | 0.0 ± 0.0 ^∗^ | 0 | 0.0 ± 0.0^∗^ | 0 | 0.0 ± 0.0^∗^ | 0 | 0.0 ± 0.0^∗^ | 0 | 0.0 ± 0.0^∗^ | 0 | 0.0 ± 0.0^∗^ |  |
| 36554 | 3N | 4.7 ± 1.1 | 3N | 4.0 ± 1.1^∗^ | 3N | 4.3 ± 0.9^∗^ | 3N | 4.7 ± 1.4^∗^ | 2N | 2.1 ± 1.4^∗^ | 2N | 3.2 ± 1.2^∗^ |  |
| *Leaves of genotypes rated without symptoms are displayed by “0,” by “0;” with occasional hypersensitive spots, by “1” with macrosocpic visible hypersensitive spots without uredospore pustules, by “2” leaves with large hypersensitive areas around uredospore pustules, by “3” with uredospore pustules and partial defense reaction on leaves, by “4” with uredospore pustules and no visible necroses or hypersensitive spots around infection sites. “N” was used if large necrotic areas were visible. Asterisks behind the averages of microscopically visible pustules per square mm of 3 replications which consist of three leaves respectively show significant differences of a near isogenic line (NIL) to the susceptible control cultivar Thatcher (α = 0.05).*  *Please note that t*he single spore isolates wxr77, isolate 167/176wxr, 13/20wxr and 58 wxr were previously kindly provided by Dr. Lind (Julius Kuehn-Institute, Quedlinburg, Germany) originated from a collection that was cultivated firstly by [Nover and Lehmann (1967)](https://www.ncbi.nlm.nih.gov/pmc/articles/PMC5101855/#B74). | | | | | | | | | | | | |  |

**Table S1B: Virulence pattern of leaf rust isolates**

| Isolates | Virulence/avirulence pattern of leaf rust isolates analysed on near isogenic lines, carrying Lr-genes singly. Cultivar Thatcher was used as susceptible control. Resistances that have been overcome are shown in front of the slash, effective resistances behind the slash in **bold letters**. Virulence/ avirulence was assessed according to the description in Table S1A where ratings from 0 to 2 were defined as avirulent and from 3 to 4 as virulent. |
| --- | --- |
| 77wxR | Lr1; Lr2a; Lr2b; Lr2c; Lr10; Lr11; Lr12; Lr13; Lr14a; Lr14b; Lr15; Lr16; Lr17; Lr18; Lr20; Lr21; Lr22a; Lr22b; Lr23; Lr28; Lr30; Lr32; Lr33; Lr34*; Lr35; Lr36; Lr37*; Lr44; Lr46; Lr49; Lr50; Lr51; Thatcher/ **Lr3a; Lr3bg; Lr3ka; Lr9; Lr19; Lr24; Lr25; Lr26; Lr27; Lr29; Lr34*; Lr39; Lr42; Lr43; Lr47; Lr48; Lr52** |
| Hk1/3-04 | Lr1; Lr2a; Lr2b; Lr2c; Lr11; Lr12; Lr13; Lr14a; Lr14b; Lr15; Lr16; Lr17; Lr18; Lr20; Lr21; Lr22a; Lr22b; Lr23; Lr26; Lr28; Lr30; Lr32; Lr33; Lr35; Lr36; Lr37; Lr40; Lr44; Lr46; Lr49; Lr41; Lr50; Lr52; Thatcher/ **Lr3a; Lr3bg; Lr3ka; Lr9; Lr10; Lr19; Lr24; Lr25; Lr27; Lr29; Lr34; Lr38; Lr39; Lr47; Lr48; Lr51;** |
| Hk12/3-01 | Lr1; Lr2a; Lr2b; Lr2c; Lr10; Lr11; Lr12; Lr13; Lr14a; Lr14b; Lr15; Lr16; Lr17; Lr18; Lr20; Lr21; Lr22a; Lr22b; Lr23; Lr26; Lr27; Lr28; Lr30; Lr32; Lr33; Lr34*; Lr35; Lr36; Lr37*; Lr39; Lr44; Lr46; Lr48; Lr49; Lr50; Lr51; Lr52; Thatcher/ **Lr3a; Lr3bg; Lr3ka; Lr9; Lr19; Lr24; Lr25; Lr29; Lr38; Lr42; Lr47** |
| 167/176WxR | Lr1; Lr2a; Lr2b; Lr2c; Lr3a; Lr3bg; Lr10; Lr11; Lr12; Lr13; Lr14a; Lr14b; Lr14b; Lr15; Lr16; Lr17; Lr18; Lr20; Lr21; Lr22a; Lr22b; Lr23; Lr27; Lr28; Lr32; Lr33; Lr35; Lr36; Lr37*; Lr44; Lr46; Lr49; Lr50; Lr51; Thatcher/ **Lr3ka; Lr9; Lr19; Lr24; Lr25; Lr26; Lr29; Lr30; Lr34; Lr38; Lr39; Lr42; Lr47; Lr48; Lr52** |
| 58WxR | Lr1; Lr2a; Lr2b; Lr2c; Lr2c; Lr3a; Lr3ka; Lr10; Lr11; Lr12; Lr13; Lr14a; Lr15; Lr17; Lr16; Lr17; Lr20; Lr22a; Lr22b; Lr23; Lr28; Lr30; Lr32; Lr33; Lr34; Lr35; Lr37; Lr44; Lr52; Thatcher/ **Lr3bg; Lr9; Lr21; Lr24; Lr25; Lr26; Lr27; Lr29; Lr36; Lr38; Lr39; Lr40; Lr45; Lr46; Lr48; Lr50; Lr51; Lr53;** |
| 13/20WxR | Lr1; Lr2a; Lr2b; Lr2c; Lr2c;B; Lr3ka; Lr10; Lr11; Lr12; Lr13; Lr14a; Lr14b; Lr15; Lr16; Lr17; Lr18; Lr20; Lr21; Lr22a; Lr22b; Lr23; Lr32; Lr33; Lr34; Lr35; Lr36; Lr37; Lr38; Lr46; Lr50; Thatcher/ **Lr3a; Lr3bg; Lr9; ; Lr19; Lr24; Lr25; Lr26; Lr27; Lr28; Lr29; Lr30; Lr39; Lr41; Lr44; Lr47; Lr48; Lr51; Lr52** |

;
