# Supplementary material for: Mapping of prehaustorial resistance against wheat leaf rust in einkorn (Triticum monococcum), a progenitor of wheat
Source: Front Plant Sci. 2023 Oct 23;14:1252123. doi: 10.3389/fpls.2023.1252123 (PMC10626456; doi:10.3389/fpls.2023.1252123)
Supplement: Supplementary file 3 [file Table_3.docx]

**Table S3: Raw phenotypic data of a the Pi272560xTb36554 crossing population**

| F2 Genotype | HMC (48hai) | HMC (72hai) | Rating score |
| --- | --- | --- | --- |
| 3_36 | 1.28 | 9.30 | 0.00 |
| 3_53 | 0.77 | 2.00 | 0.00 |
| 3_68 | 1.04 | 8.90 | 1.00 |
| 1_17 | 1.73 | 9.80 | 0.00 |
| 1_30 | 0.77 | 1.60 | 0.00 |
| 1_49 | 1.00 | 4.10 | 2.00 |
| 1_64 | 0.93 | 5.40 | 0.00 |
| 2_20 | 0.83 | 6.40 | 0.00 |
| 2_29 | 0.40 | 2.50 | 0.00 |
| 3_1 | 4.43 | 2.80 | 0.00 |
| 3_22 | 1.30 | 10.86 | 0.00 |
| 3_37 | 1.35 | 17.80 | 0.00 |
| 3_54 | 0.58 | 12.80 | 0.00 |
| 3_72 | 0.72 | 6.30 | 0.00 |
| 1_18 | 2.13 | 7.30 | 1.00 |
| 1_32 | 2.07 | 9.90 | 1.00 |
| 1_50 | 1.73 | 12.00 | 0.00 |
| 2_6 | 2.24 | 10.00 | 2.00 |
| 2_22 | 0.60 | 8.70 | 0.00 |
| 2_32 | 1.41 | 6.20 | 0.00 |
| 3_3 | 0.69 | 16.80 | 0.00 |
| 3_24 | 0.86 | 12.10 | 0.00 |
| 1_1 | 1.55 | 2.30 | 0.00 |
| 3_38 | 1.89 | 4.90 | 0.00 |
| 3_55 | 0.83 | 10.10 | 0.00 |
| 3_76 | 1.00 | 5.20 | 0.00 |
| 1_20 | 1.77 | 6.70 | 2.00 |
| 1_38 | 1.30 | 5.50 | 0.00 |
| 1_54 | 1.03 | 6.40 | 2.00 |
| 2_7 | 1.67 | 4.90 | 0.00 |
| 2_23 | 3.07 | 11.00 | 0.00 |
| 2_33 | 2.57 | 8.80 | 1.00 |
| 3_6 | 1.03 | 15.60 | 0.00 |
| 3_27 | 2.37 | 9.40 | 2.00 |
| 1_5 | 0.93 | 2.80 | 0.00 |
| 3_41 | 1.23 | 7.80 | 0.00 |
| 3_57 | 0.65 | 3.60 | 0.00 |
| 3_77 | NA | 2.20 | 0.00 |
| 1_21 | 1.67 | 6.70 | 1.00 |
| 1_39 | 1.43 | 6.60 | 0.00 |
| 1_55 | 0.53 | 1.60 | 0.00 |
| 2_8 | 0.18 | 4.30 | 0.00 |
| 2_38 | 1.97 | 7.50 | 0.00 |
| 3_8 | 0.37 | 3.40 | 0.00 |
| 3_29 | 1.07 | 12.10 | 0.00 |
| 1_6 | 1.00 | 2.20 | 0.00 |
| 3_46 | 0.67 | 11.90 | 1.00 |
| 3_60 | 0.80 | 2.40 | 2.00 |
| 3_79 | NA | 9.70 | 0.00 |
| 1_22 | 0.93 | 8.70 | 0.00 |
| 1_41 | 0.53 | 9.30 | 0.00 |
| 1_58 | 0.93 | 2.30 | 0.00 |
| 2_13 | 0.87 | 10.30 | 0.00 |
| 2_25 | 0.57 | 7.80 | 0.00 |
| 2_39 | 1.07 | 10.40 | 0.00 |
| 3_11 | 1.13 | 8.50 | 0.00 |
| 3_30 | 1.33 | 10.60 | 1.00 |
| 1_7 | 1.10 | 2.80 | 0.00 |
| 3_47 | 0.89 | 5.80 | 0.00 |
| 3_61 | 1.17 | 1.90 | 0.00 |
| 4_1 | NA | 6.80 | 0.00 |
| 1_24 | 1.47 | 2.20 | 0.00 |
| 1_42 | 0.63 | 7.00 | 0.00 |
| 1_59 | 1.80 | 13.20 | 0.00 |
| 2_14 | 1.77 | 6.60 | 0.00 |
| 2_26 | 0.53 | 2.50 | 0.00 |
| 2_40 | 3.10 | 2.00 | 0.00 |
| 3_14 | 0.55 | 5.80 | 0.00 |
| 3_31 | 4.59 | 8.70 | 1.00 |
| 1_8 | 1.20 | 2.71 | 0.00 |
| 3_49 | 5.23 | 6.60 | 0.00 |
| 3_66 | 2.07 | 8.40 | 0.00 |
| 1_26 | 0.51 | 2.20 | 0.00 |
| 1_43 | 2.23 | 7.80 | 0.00 |
| 1_61 | 2.20 | 7.00 | 0.00 |
| 2_16 | 2.50 | 6.60 | 1.00 |
| 2_27 | 0.27 | 9.70 | 0.00 |
| 2_48 | 0.46 | 7.60 | 0.00 |
| 3_16 | 3.23 | 8.40 | 0.00 |
| 3_32 | 1.47 | 1.70 | 0.00 |
| 1_11 | 0.50 | 2.20 | 0.00 |
| 3_50 | 1.27 | 10.50 | 1.00 |
| 3_67 | 2.77 | 11.20 | 0.00 |
| 1_27 | 0.50 | 0.60 | 0.00 |
| 1_47 | 2.00 | 6.90 | 0.00 |
| 1_63 | 2.13 | 6.50 | 0.00 |
| 2_18 | 3.47 | 9.30 | 2.00 |
| 2_28 | 2.70 | 9.10 | 0.00 |
| 2_50 | 0.70 | 15.90 | 0.00 |
| 3_21 | 0.50 | 2.40 | 0.00 |
| 3_35 | 0.37 | 1.50 | 0.00 |
| 5_18 | 4.07 | 2.60 | 0.00 |
| 5_41 | 3.73 | 1.50 | 0.00 |
| 5_69 | 1.37 | 1.00 | 0.00 |
| 4_16 | 0.83 | 3.00 | 0.00 |
| 4_33 | 0.93 | 8.40 | 0.00 |
| 4_45 | 1.10 | 1.40 | 0.00 |
| 4_56 | 1.13 | 7.60 | 0.00 |
| 4_73 | 0.50 | 1.80 | 1.00 |
| 4_87 | 6.60 | 5.70 | 0.00 |
| 4_96 | 4.77 | 1.10 | 2.00 |
| 5_5 | 1.93 | 1.20 | 0.00 |
| 5_22 | 1.80 | 8.40 | 1.00 |
| 5_42 | 2.80 | 2.00 | 0.00 |
| 5_70 | 2.97 | 6.80 | 0.00 |
| 4_17 | 1.27 | 2.50 | 0.00 |
| 4_34 | 1.37 | 1.20 | 0.00 |
| 4_47 | 0.97 | 1.50 | 0.00 |
| 4_57 | 1.30 | 7.10 | 0.00 |
| 4_76 | 1.40 | 1.11 | 0.00 |
| 4_88 | 2.53 | 3.67 | 2.00 |
| 4_101 | 4.43 | 5.40 | 0.00 |
| 5_7 | 2.40 | 1.60 | 0.00 |
| 4_3 | NA | 2.00 | 0.00 |
| 5_27 | 2.40 | 0.60 | 0.00 |
| 5_45 | 7.93 | 1.40 | 0.00 |
| 5_71 | 2.97 | 5.50 | 0.00 |
| 4_20 | 1.10 | 2.00 | 0.00 |
| 4_35 | 0.90 | 0.90 | 0.00 |
| 4_48 | 2.67 | 2.30 | 0.00 |
| 4_58 | 1.70 | 2.40 | 0.00 |
| 4_77 | 1.10 | 1.78 | 1.00 |
| 4_89 | 3.43 | 7.67 | 0.00 |
| 4_102 | 3.20 | 5.50 | 0.00 |
| 5_8 | 1.80 | 2.70 | 0.00 |
| 4_4 | 1.30 | 1.20 | 0.00 |
| 5_28 | 2.60 | 1.50 | 0.00 |
| 5_46 | 1.33 | 1.60 | 1.00 |
| 5_73 | 3.37 | 2.30 | 1.00 |
| 4_21 | 0.83 | 2.10 | 0.00 |
| 4_37 | 2.10 | 1.20 | 0.00 |
| 4_50 | 2.97 | 1.80 | 0.00 |
| 4_61 | 0.90 | 1.50 | 0.00 |
| 4_79 | 0.93 | 2.10 | 0.00 |
| 4_90 | 1.30 | 1.78 | 0.00 |
| 4_103 | 4.43 | 1.90 | 0.00 |
| 5_9 | 4.57 | 1.50 | 0.00 |
| 4_5 | 1.37 | 1.60 | 0.00 |
| 5_29 | 3.23 | 1.90 | 0.00 |
| 5_47 | 5.07 | 4.70 | 2.00 |
| 5_74 | 4.83 | 2.00 | 0.00 |
| 4_27 | 1.23 | 7.90 | 0.00 |
| 4_39 | 1.90 | 1.90 | 0.00 |
| 4_51 | 0.87 | 2.20 | 0.00 |
| 4_62 | 1.03 | 1.10 | 2.00 |
| 4_83 | 2.23 | 1.50 | 0.00 |
| 4_91 | 4.70 | 7.67 | 0.00 |
| 4_105 | 1.70 | 1.60 | 0.00 |
| 5_10 | 2.97 | 8.83 | 0.00 |
| 4_7 | 0.97 | 2.40 | 0.00 |
| 5_32 | 1.96 | 6.40 | 0.00 |
| 5_49 | 5.80 | 1.70 | 0.00 |
| 5_77 | 5.93 | 2.50 | 0.00 |
| 4_28 | 0.93 | 1.80 | 0.00 |
| 4_40 | 1.90 | 2.30 | 0.00 |
| 4_53 | 1.23 | 2.10 | 0.00 |
| 4_66 | 2.77 | 2.10 | 0.00 |
| 4_84 | 1.43 | 5.10 | 0.00 |
| 4_92 | 0.93 | 7.89 | 0.00 |
| 5_2 | 2.33 | 1.11 | 0.00 |
| 5_12 | 3.97 | 2.10 | 0.00 |
| 4_10 | 1.40 | 2.20 | 0.00 |
| 5_34 | 1.77 | 2.10 | 0.00 |
| 5_60 | 5.80 | 1.60 | 0.00 |
| 4_30 | 1.93 | 7.20 | 0.00 |
| 4_41 | 1.23 | 1.60 | 2.00 |
| 4_54 | 2.10 | 2.40 | 0.00 |
| 4_69 | 0.47 | 7.80 | 1.00 |
| 4_85 | 1.82 | 1.10 | 0.00 |
| 4_93 | 8.67 | 1.20 | 0.00 |
| 5_13 | 6.03 | 7.00 | 0.00 |
| 4_14 | 1.77 | 2.30 | 0.00 |
| 5_36 | 4.50 | 1.80 | 0.00 |
| 5_67 | 2.80 | 2.10 | 1.00 |
| 4_31 | 2.40 | 8.00 | 1.00 |
| 4_44 | 2.60 | 1.30 | 0.00 |
| 4_55 | 1.53 | 1.50 | 1.00 |
| 4_70 | 3.40 | 8.56 | 0.00 |
| 4_86 | 2.10 | 5.50 | 0.00 |
| 4_94 | 2.03 | 2.40 | 1.00 |
| 5_4 | 2.13 | 0.80 | 0.00 |
| 5_17 | 2.87 | 1.60 | 0.00 |

* Please note: HMC = Haustorial mother cells, hai = hours after inoculation, The phenotypic data was obtained as described previously in the material and methods section of the manuscript.
